# Genomic Prediction for Twin Pregnancies

**DOI:** 10.3390/ani11030843

**Published:** 2021-03-16

**Authors:** Shaileen P. McGovern, Daniel J. Weigel, Brenda C. Fessenden, Dianelys Gonzalez-Peña, Natascha Vukasinovic, Anthony K. McNeel, Fernando A. Di Croce

**Affiliations:** 1 Zoetis Genetics, 333 Portage Street, Kalamazoo, MI 49007, USA; shaileen.mcgovern@zoetis.com (S.P.M.); brenda.fessenden@zoetis.com (B.C.F.); Di.Gonzalez@zoetis.com (D.G.-P.); natascha.vukasinovic@zoetis.com (N.V.); anthony.mcneel@zoetis.com (A.K.M.); 2 Zoetis Outcomes Research, 333 Portage Street, Kalamazoo, MI 49007, USA; daniel.j.weigel@zoetis.com

**Keywords:** genetics, genomics, twinning, Holstein, selection index, breeding strategy, prediction, STA

## Abstract

**Simple Summary:**

Twinning in dairy cattle is caused by many different factors, both genetic (i.e., inherited) and non-genetic (i.e., animal management). In dairy operations, twinning is an undesirable trait associated with other reproductive and metabolic diseases, higher operational costs, and higher rates of culling on farm. The animal welfare and economic impacts have resulted in the development of a genomic prediction for twinning (i.e., TWIN) by Zoetis such that producers can make informed breeding decisions for breeding Holstein females that are less likely to become pregnant with twins in a given lactation. This prediction is included in a holistic breeding tool (i.e., selection index) for producers so that they can improve multiple health, fertility, and production traits in parallel with reducing twinning when making breeding decisions for future generations. The objectives of the present study were (1) to describe how the twinning prediction was developed (and included in a selection index), (2) show that the prediction works effectively using real life farm data, and (3) propose how this genetic tool can be used in collaboration with management practices to proactively reduce twin pregnancies on farm. The results of this study provide evidence that twinning can be proactively managed on dairy farms using genetically powered tools.

**Abstract:**

Twinning is a multifactorial trait influenced by both genetic and environmental factors that can negatively impact animal welfare and economic sustainability on commercial dairy operations. To date, using genetic selection as a tool for reducing twinning rates on commercial dairies has been proposed, but not yet implemented. In response to this market need, Zoetis (Kalamazoo, MI, USA) has developed a genomic prediction for twin pregnancies, and included it in a comprehensive multitrait selection index. The objectives of this study were to (1) describe a genetic evaluation for twinning in Holstein cattle, (2) demonstrate the efficacy of the predictions, (3) propose strategies to reduce twin pregnancies using this information. Data were retrieved from commercial dairies and provided directly by producers upon obtaining their permission. The twin pregnancies trait (TWIN) was defined as a pregnancy resulting in birth or abortion of twin calves, classified as a binary (0,1) event, and analysed using a threshold animal model. Predictions for a subset of cows were compared to their on-farm twin records. The heritability for twin pregnancies was 0.088, and genomic predicted transmitting abilities ((g)PTAs) ranged from −7.45–20.79. Genetic correlations between TWIN and other traits were low, meaning that improvement for TWIN will not negatively impact improvement for other traits. TWIN was effectively demonstrated to identify cows most and least likely to experience a twin pregnancy in a given lactation, regardless of reproductive protocol used. Effective inclusion of the prediction in a multitrait selection index offers producers a comprehensive tool to inform selection and management decisions. When combined with sound management practices, this presents a compelling opportunity for dairy producers to proactively reduce the incidence of twin pregnancies on commercial dairy operations.

## 1. Introduction

Twinning in cattle is a complex trait that can be influenced by a multitude of factors such as milk production, season, breed, parity and ovulation rate, previous twin calving events, pharmaceutical use, and genetics [1,2,3,4,5,6,7,8]. Although generally perceived to be a positive reproductive outcome in the beef industry (due to increased operational efficiencies in terms of increased weaned calf weight per dam [9,10]), twinning is a negative reproductive outcome for dairy operations, as it adversely affects cow and calf health and welfare, and ultimately reduces herd profitability [11,12]. Despite cattle being a monotocous species, twinning rates in cattle range from <1% in beef populations up to 5.8% in dairy populations, with appreciably high twinning rates observed in the Holstein breed, relative to other cattle breeds [12,13,14,15,16]. The deleterious health effects of twinning are numerous and include increased incidences of mortality, stillbirths and abortions, and reduced birth weights for calves [2,17,18,19]. For cows, higher incidences of periparturient diseases such as retained placenta, metritis, displaced abomasum, and ketosis are associated with twin-bearing cows; this is all in addition to a reduced overall productive lifespan for these cows [2,20,21,22,23]. Moreover, twinning also negatively impacts reproductive performance of cows and calves alike, resulting in increased days open, increased services per conception, cystic ovarian disease and dystocia for cows, and freemartinism in most heifer calves twinned to bull calves [1,2,4,21,24,25]. Taking this myriad of detrimental effects into account, twinning increases veterinary, culling and replacement heifer costs on farm, thus eroding profit margins and impacting operational sustainability. Previous estimations of the economic ramifications of twinning range from $97–$225 per twin pregnancy on farm, with an overall estimated annual impact on US dairy profitability ranging between $22.5–$112.5 million [16,26,27,28,29]. These health, welfare and economic challenges imposed by twinning on the industry are projected to rise, as selecting for higher milk output has previously been associated with higher rates of twinning, and the aim of dairy operations is to maximize milk production output per cow [1,2].

Once diagnosed, current intervention and management practices for twin pregnancies include (1) GnRH treatment to maintain the gestation, (2) induced embryo reduction through aspiration of the allanto-amniotic fluid or manual rupture, (3) induced abortion using PGF_2α_, and (4) culling. Limitations of these methods include an increased likelihood of pregnancy loss in the embryo that was not selected for manual reduction, and in the case of pregnancy maintenance, the aforementioned periparturient diseases and abortion, stillbirth, calf mortality, and dystocia [11,12,27,30,31]. Opinions differ on what is the optimal way to manage females carrying twin calves, with some advocating manual embryo reduction [27], and others suggesting selective culling and selective maintenance of pregnancy, depending on the value of the cow and calf(ves) as the preferred alternative [12]. Since prior twin pregnancy diagnosis/calving events is a risk factor for future twinning events, these methods are somewhat short-sighted and do not help reduce the prevailing trend in twin pregnancies in the industry in a proactive manner. Importantly, regardless of which management decision is ultimately chosen, all of these methods are reactive in nature. Alternative, proactive solutions, such as those that use genetics and genomics to predict the likelihood of females to be diagnosed with twins in a given lactation are warranted, as the trend toward increased twinning in the dairy cattle population is ever increasing, given the industry selects for higher-output cattle [12].

There is an established historical and ongoing effort by research groups across the globe to quantify the genetic component of twinning in cattle. Notably, since the 1980′s the USDA Meat Animal Research Center (USDA-MARC) has quantified the genetic parameters of, and identified areas in the bovine genome associated with twinning in cattle, in addition to demonstrating the effectiveness of selective breeding for twinning in cattle based on their observed twinning rates [7,8,21,32]. Twinning rates in this research herd have exceeded 50%, and a proportion of the foundation herd was reported to be comprised of Holstein genetics (with just under one third of the genetic makeup of the population tracing back to the Holstein) [7,33]. Heritability estimates reported globally for twinning in dairy cattle range from 0.004–0.29, and vary depending on the extent of variability (residual and genetic) in the populations sampled, sample sizes, and model used (i.e., linear, threshold, animal, sire) [7,8,13,16,29,32,34,35,36,37,38,39,40,41]. These non-zero estimates, alongside the reported genetic variation in twinning highlights that there is ample opportunity for selection and breeding decisions to reduce the frequency of twinning events in the dairy population. Quantitative trait loci (QTLs, i.e., regions in the genome) previously identified as being associated with twinning in cattle further substantiates the existence of a genetic and genomic component to twinning, with QTLs identified on *Bos taurus* autosome 1, 5, 6, 7, 8, 10, 14, 15, 19, 23, and 24 [39,41,42,43,44,45,46,47,48,49,50,51].

Recent advancements in implementing hormonal synchronization protocols which proactively reduce the incidence of double ovulation in dairy cattle has shown to be an effective non-genetic (i.e., environmental) tool in this space [30]. However, in spite of the relatively large body of work investigating the genetics(omics) underpinning twinning, Fricke [12] stated that “at present, dairy herders and their consultants are ill prepared to make sound management decisions to mitigate the negative effects of twinning on their operations because of a lack of basic and applied scientific data on twinning in dairy cattle”. This statement has largely held true from a genomics perspective, due to the relative lack of industry-available predictive tools for producers to proactively reduce twinning rates on their dairy operations globally. To date, there have been a number of studies that conducted genetic(omic) evaluations for twinning rate in cattle [16,35,37,39,40,52], however these evaluations have not been translated in to predictions for twinning that producers in the industry can use to proactively select for cows less likely to experience a twin pregnancy in a given lactation. Using genetic selection as a tool to reduce on-farm twinning rates has been researched, yet, so far, has not been implemented. Genomic predictions for twinning events can provide the foundation needed to make proactive and informed breeding and management decisions to reduce their frequency, provided they are included in a comprehensive, economically driven multitrait selection index tool.

Harnessing such genetic- and genomically powered tools, in tandem with practical and strategic breeding and management strategies can holistically offer producers the ability to proactively reduce the incidence of twinning in their herds, helping improve animal wellbeing, economic outcomes [53,54], and peace of mind. Selection indices are tools that condense information on the genetic merit of animals across multiple traits in to one value, allowing a producer to easily rank animals and subsequently create breeding and management strategies to maximize genetic gain in the herd toward their selection goal(s) [55,56]. Historically, the goal of selection indices was to focus solely on improving production traits [57,58], to the detriment of cow fertility and health traits arising from the antagonistic relationships that exist between these traits [54,59]. The appreciation of these antagonistic relationships, along with the shift to not only focusing on traits that increase on-farm profit, but those that decrease costs of production has resulted in the inclusion of these non-production traits in multitrait selection indices globally [56,60].

In response to industry needs for genetic improvement of dairy wellness traits, Zoetis Genetics developed an industry-available genetic and genomic evaluation for wellness traits in Holstein cattle in 2016, known as CLARIFIDE^®^ Plus (Zoetis Genetics, Kalamazoo, MI, USA). CLARIFIDE^®^ Plus provides producers with a comprehensive suite of genomic predictions, plus a multitrait selection index tool, the Dairy Wellness Profit^TM^ (DWP$) index to facilitate sound management and selection decisions based on the genetic predisposition of their heifer calves for these traits that will be expressed later in life. DWP$ is an economic multitrait selection index that was formulated to estimate the potential lifetime profitability an animal would generate under US dairy economic conditions and includes cow and calf wellness, production, fertility, functional type, longevity, livability, calving ability, and milk quality traits, as well as polled test results [56,61]. In 2018 and 2020, CLARIFIDE^®^ Plus and DWP$ were updated to include additional traits shown to impact the lifetime profitability of a dairy animal; one of those traits included in the 2020 update was twinning (i.e., TWIN). The objective of the present study was to (1) describe a genetic and genomic evaluation for twinning in Holstein cattle (including how this information is incorporated into a comprehensive and easy to interpret multitrait selection index), (2) demonstrate the ability of the evaluation’s predictions to accurately predict twinning incidences (regardless of on-farm reproductive protocol used), and (3) propose practical ways to holistically apply this information to offer a proactive solution that producers can implement to reduce the incidence of twinning in Holstein dairy herds.

## 2. Materials and Methods

### 2.1. Data Sources for Genetic Evaluation

Data was available from 276 herds located in 26 states in the US and collected approximately from 1990 to date. Each herd supplied, on average 22,998 phenotypic records. Data were obtained directly from producers upon obtaining their signed permissions; the herds were not routinely monitored or compensated for event recording by Zoetis. Production, reproduction, and pedigree information was extracted from on-farm herd management software backup files using proprietary scripts. 

Genotypes were obtained from the Zoetis Genotyping Lab (Zoetis Genetics, Kalamazoo, MI, USA). Animals were genotyped with a variety of low-density SNP chips with a number of SNPs ranging from about 3000 to over 35,000, and several types of medium-density chips with 50,000–80,000 SNPs. All animals genotyped on chips with <40,000 SNPs were imputed using the program FImpute [62] to 45,245 SNPs used in the genomic evaluation. 

### 2.2. Data Editing and Genetic Trait Definition for Genetic Evaluation

Initial data editing included checking animal identification for accuracy and consistency across data files, using similar criteria as described in Norman et al. (1994) [63]. Each animal was required to have a lactation record with a valid calving date and a lactation number as well as a calving interval between 250 and 999 days. Phenotype records were checked against the pedigree and all animals found to be male in the pedigree file or having a calving date preceding their birth date were removed.

The genetic trait of twin pregnancies (i.e., TWIN) was defined as a pregnancy resulting in birth or abortion of twin calves (alive or dead). TWIN was treated as a binary event-having a value of one if the cow was recorded as carrying or giving birth to twins and zero otherwise. Records of the same cow without TWIN recorded, as well as records of all herdmates without recorded twins, were added as ‘singleton pregnancy’ records (i.e., ‘0’). Twinning information was extracted from a combination of the events and remarks recorded on the herd management software. Terminology used to record TWIN events varied across the herds, which were standardized and collapsed into the binary TWIN outcome as shown Table 1.

Further, each herd by year and season of calving (HYS) group was required to have a minimum of 20 records and at least one recorded twinning event. HYS groups that did not meet these criteria were omitted from analysis, as it was assumed that the herd did not record twinning at all, or did not record it during that time period.

### 2.3. Statistical Models for Genetic Evaluation

The analyses for the present study were conducted largely as described by Gonzalez-Peña et al. (2020) [64]; the following threshold animal model with repeated observations was used to conduct the analysis:

*λ* = *Xβ* + *Z_h_h* + *Z_a_a* + *Z_p_p* + *e* (1)

where *λ* represents a vector of unobserved liabilities to twinning; *β* is the vector of fixed parity effects, with the corresponding incidence matrix *X*; parities 1, 2, 3, 4, and ≥5 were considered; *h* is the random herd-year-season effect, where *h*~*N*(0,*Iσ*_*h*_^2^), with the variance *σ_h_*^2^. Four seasons were defined within each calving year: Winter (December–February), Spring (March–May), Summer (June–August), and Fall (September–November); *a* is the random animal effect with *a*~*N*(0,*Hσ*_*a*_^2^), where *σ*_*a*_^2^ is the additive genetic variance and H is the pedigree relationship matrix augmented using genotypes; *p* is the random effect of permanent environment with *p*~*N*(0,*Iσ*_*p*_^2^), with *σ*_*p*_^2^ is the permanent environment variance and *e* is the residual, where *e*~*N*(0,*I*).

Variance components were estimated using the same model, but without genotypes using the program THRGIBBS1F90 version 2.108 from the BLUPF90 family [65]. Genetic evaluation was performed using the programs from the BLUPF90 family [66]. A univariate threshold model based on single step genomic BLUP methodology (ssGBLUP) was applied. The inverse of the traditional pedigree relationship matrix, 𝐴^−1^, was replaced by the inverse of H matrix that combines pedigree and genomic relationships [67,68]:

(2) $$H^{-1}=A^{-1}\;+\;\left[\begin{array}{cc} 0 & 0\\ 0 & G^{-1}-A_{22}^{-1} \end{array}\right]$$

where 𝐴^−1^ is an inverse of the pedigree relationship matrix, 𝐺^−1^ is an inverse of the genomic relationship matrix and 𝐴_22_^−1^ is an inverse of the pedigree-relationship matrix for genotyped animals only. The ‘algorithm for proven and young animals’ (APY) developed by Ignacy Misztal’s group from the University of Georgia in Athens (UGA, Georgia) was applied. The program CBLUP90IOD2 version 3.39 was used to obtain genomic breeding values using preconditioned conjugate gradient (PCG) with the number of rounds set to 200. The core consisted of 25,000 randomly selected animals. The genomic matrix conditioning parameters tau and omega were set to 1.0. Inbreeding was considered when constructing the pedigree relationship matrix. The reliabilities of estimated breeding values (EBVs) were obtained with the program ACCF90GS version 2.54, which approximates reliabilities using contribution from genotypes, phenotypes, and pedigree. To reduce computational requirements, the contribution from genotypes is replaced by the value of the diagonal of the G matrix, *g_ii_*. Reliabilities for genotyped animals were approximated as per Gonzales-Peña et al. (2020) using the guidance of Daniela Lourenco, University of Georgia, Athens, personal communication, 2016 [69].

The solutions from the CBLUP90IOD2 program (raw EBVs) were transformed into probabilities of exceeding the threshold value, with threshold values being calculated from the current data. For each animal solution, we calculated probability that a standard normal variable with the mean equal to this solution and the variance of one exceeds the threshold. The probabilities were then multiplied by 100 (to represent percent), divided by 2 (to obtain predicted transmitting abilities (PTAs)), and expressed as the deviation from the average PTA of all animals (genotyped or not (i.e., having a gPTA or a PTA)) born in 2015 with a phenotypic record for TWIN. The resulting (g)PTAs may be interpreted as differences of the individual animal’s risk of having twins from the average (base) risk. Higher values of (g)PTAs indicate a higher chance of twinning. For example, an animal with a TWIN (g)PTA of −2.0 has a 2% lower risk, whereas an animal with a TWIN (g)PTA of 2.0 has a 2% higher risk of having twins in a given lactation than the base animal [64]. For ease of interpretation, (g)PTAs were transformed into standardized transmitting abilities (STAs), with a mean of 100, a standard deviation of 5, and the reversed sign (so that higher values represent lower risk of TWIN) as per McNeel et al. (2017) [54,64]. Correlations between TWIN STAs and other traits (Zoetis Wellness trait STAs and trait (g)PTAs in the US genetic evaluation (Council of Dairy Cattle Breeding)) were estimated using product-moment (Pearson) correlations, similar to Gonzalez-Peña et al. (2020) [64].

### 2.4. Inclusion of Twinning Prediction in a Multitrait Selection Index

An in-depth description of the development of DWP$ is detailed in Fessenden et al. (2020) [56]. Briefly for the purposes of this study, (1) the TWIN STA, its phenotypic correlations, and genetic relationships with other traits were estimated, (2) the economic value of the TWIN trait as it relates to its contributions to a dairy animal’s lifetime profitability was estimated (by determining the economic value of all incomes and losses for a 12-unit increase in the TWIN trait), and (3) the STA for TWIN is multiplied by its corresponding economic weight, alongside all other traits in the DWP$ index, which are then summed together to determine an animal’s overall selection index value [56,70].

### 2.5. Demonstration of Evaluation Efficacy

To demonstrate the efficacy of the TWIN predictions generated by the evaluation, a subset of 8,219 females from 5 US herds that used commercial genomic tests were selected, and the relationship between the TWIN phenotype and genetic(omic) merit for TWIN investigated for each herd. Criteria for inclusion in these demonstration cohorts included (1) the animals/herds did not contribute phenotypes to the genetic evaluation so as to not bias results, (2) the herds demonstrated adequate recording of TWIN events on a per-animal basis, and (3) a single reproductive protocol was required to be used consistently on farm (*n* = 3, defined as: no synchronization protocol, presynch ovsynch protocol, or Double Ovsynch protocol). Table 7 outlines the overview 5 herds used in the demonstration cohort.

Once selected, TWIN STAs for these animals were adjusted to reflect what their STA values would have been in 2013. This measure was included to reduce bias in the TWIN estimate predictions such that the predictions closely reflect what the producer would have received before the animals entered the milking string. This measure reduces the bias that could arise from having 2014–2020 records (pedigree and performance) feeding into the evaluation. Females were then ranked based on TWIN STA predictions within each herd, and allocated to one of three genetic groups (i.e., tertiles): worst 33%, 34–66%, and best 33%, similar to what has been reported by others [54,56,71]. Data analysis was generated using SAS software (version 9.4, SAS Institute Inc., Cary, NC, USA). For this analysis, differences between genetic groups were considered to be statistically significant when *p*-value < 0.05. The binary TWIN events (0,1) for each herd were analyzed using PROC GLIMMIX with a binomial distribution and a logit link function using the following model:

Y = Xβ + Zμ + e(3)

where Y represents the vector of the TWIN phenotype; β represents the fixed effects of TWIN genetic group (worst 33%, 34–66%, best 33%) and lactation (2nd–4th); μ represents the random effects of animal nested within lactation to account for repeated measures; and e represents the residual, with X and Z design matrices relating observations Y to β and μ, respectively. Marginal means (i.e., twinning incidence), the standard error of the mean, and the *p*-values are reported. Finally, cost per cow per genetic group for TWIN was calculated as per McNeel et al. (2017) [54], using the average of the most and least conservative estimates ($97 and $225, respectively) developed in a comprehensive economic analysis by Mur-Novales et al. (2018) [27]. A total of 6280 females were analysed.

Secondarily, the association of individual animal TWIN STAs, lactation and TWIN outcomes was estimated as supporting data, with the model’s β term updated to represent the fixed effects of TWIN STA (85–115 inclusive, i.e., ±3SD) and lactation (1st–4th), and μ updated to represent the random effect of animal. Plots of the association between the individual animal STAs and TWIN outcome are reported.

Tertiarily, the association between TWIN STA, season of conception (spring, summer, autumn, winter), and peak lactation output (from the previous lactation) with TWIN outcome was estimated as supporting data. This was completed given previous reports of associations between season of conception and peak lactation with TWIN [1,2,3,5]. In this analysis, the model was updated such that the β term represented the fixed effects of TWIN STA, season of conception, and peak lactation output of the previous lactation. This tertiary analysis was completed using 3rd lactation records for the 5 herds. This lactation was chosen given TWIN incidence was appreciable, peak lactation data from the previous lactation was available, and culling bias was relatively low. Type III Tests of Fixed Effects for TWIN STA, season of conception and peak lactation output of the previous lactation are reported. A total of 4,162 females were analysed.

## 3. Results

### 3.1. Data Characteristics for Genetic Evaluation

The total number of pedigree records, phenotypic records, animals with phenotypes, animals with genotypes, animals with both genotypes and phenotypes, and the incidence of twinning as of August 2020 are given in Table 2.

### 3.2. Variance Components and Summary Statistics for Genetic Evaluation

Table 3 shows the estimated variance components for twinning in Holsteins. The estimated heritability of twinning was 0.088, with a repeatability of 0.18.

**Figure 1:**
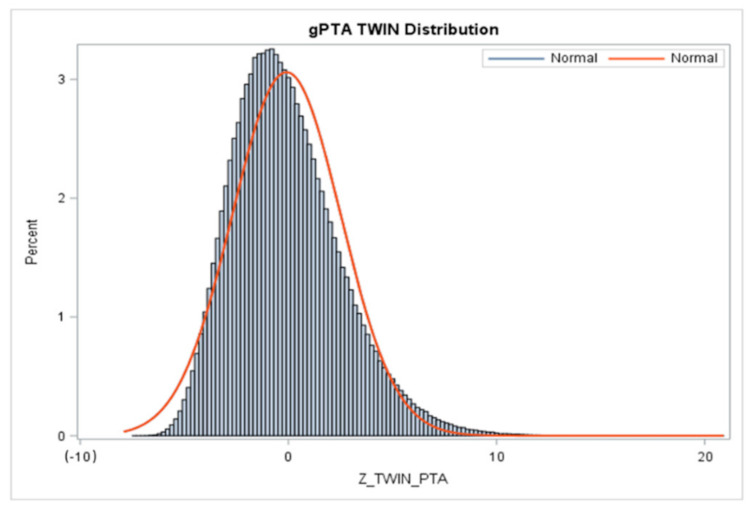
Distribution of (g)PTAs (Z_TWIN_PTA) for twinning for all animals in the Holstein evaluation. An appreciable variation in (g)PTAs is observed in the Holstein population sampled in the present study.

The means, standard deviations, minimum and maximum of (g)PTAs, STAs, and reliabilities for TWIN are presented in Table 4. Figure 1 shows the associated distribution of (g)PTAs for twinning.

### 3.3. Correlations of Twinning with Other Traits

Table 5 and Table 6 show the product-moment (Pearson) correlations of genomic predictions for TWIN with predictions for the DWP$ index and other wellness traits offered in CLARIFIDE^®^ Plus, as well as the correlations of TWIN with economically important traits in the genetic evaluation produced by the Council on Dairy Cattle Breeding (CDCB). As expected, the strongest correlations exist between TWIN and RETP, ABORT and METR. The weak correlation between TWIN and production traits communicate that making genetic progress for TWIN would not negatively impact genetic progress for those production traits, and vice versa.

### 3.4. Demonstration of Evaluation Efficacy

An overview of the herd data is presented in Table 7, showing an incidence for first and third lactation per herd. First lactation incidences range from 0.56–1.48%, and third lactation incidences range from 6.95–14.00%.

Differences in twinning incidence (marginal means) were statistically significant between the genetic groups (*p* < 0.0001). As shown in Table 8, the differences in twinning incidence between the top and bottom tertiles was 9%, 5%, 6%, 6%, and 7% for herd 1, 2, 3, 4, and 5 respectively. 

Previously published disease economic costs demonstrate that the differences in marginal means by genetic groups (disease incidence) translate into appreciable differences in expected economic costs on a US dairy operation (Table 8). Cows in the lowest genetic risk group (Best 33%) for Twinning (i.e., highest TWIN STAs) had 9%, 5%, 6%, 6%, and 7% percentage points lower frequency of twinning for herd 1, 2, 3, 4, and 5 respectively, which represents a 47%, 56%, 50%, 67%, and 78% difference in incidence of twinning when compared to the highest risk group (Worst 33%). This translates into decreased losses per cow of $15, $8, $9, $9 and $11 for herd 1, 2, 3, 4, and 5 respectively.

Table 9 reports the significance for each of the fixed effects included in the tertiary model, with *p*-values ranging from 0.0033 (TWIN STA 2013 for herd 5) to 0.9129 (season of conception for herd 1). TWIN STA was a significant predictor of TWIN for 4 out of the 5 herds, with the exception of herd 2 (*p*-values ranging from 0.0033–0.03 for herds 1, 3, 4 and 5, and 0.1147 for herd 2). Season of conception was not a significant predictor for any herds (*p*-values ranging from 0.2779–0.9129). Peak lactation (previous lactation) was a significant predictor for TWIN for 1 of the 5 herds, with a *p*-value of 0.0495 for herd 3 (and ranging from 0.0650–0.8849 for the other herds).

## 4. Discussion

The incidence of twinning records in the genetic evaluation of 3.25% reported here is similar to incidences reported in previous studies, which range from <1–5.8% [12,13,14,15,16,37]. Reported estimates in this study for heritability, repeatability, genetic correlations and (g)PTAs are consistent with those previously published [7,8,13,16,29,32,34,35,36,37,38,39,40,41]. To the best of our knowledge, this is the first time a genetic and genomic evaluation for TWIN in female dairy cattle has been described and demonstrated to predict actual twinning events using producer-recorded data, and subsequently included in a holistic multitrait selection index. The observed efficacy of using producer-recorded data for conducting successful genetic evaluations of dairy cow wellness traits corroborates the results of previous studies [53,61,64,72]. Given the demonstrated variation in, and efficacy of TWIN STA predictions observed in this study, in addition to the economic and animal health-related impacts of TWIN, the justification for including a genomic prediction for twin pregnancies in a balanced multitrait selection index is strong. That being said, truly efficacious protocols for reducing the incidence of TWIN on farm will arise from combining genetic (omic) tools and best practices previously documented [11,12,27,30], examples of which are outlined in Section 4.4 below. 

### 4.1. Variance Components and Genetic (omic) Correlations

Previous studies that estimated genetic parameters for twinning in cattle suggest that heritability estimates for the trait range from 0.004–0.29, with repeatabilities (where reported) ranging from 0.04–0.55 [7,8,13,16,29,32,34,35,36,37,38,39,40,41]. The heritability for TWIN reported in this study is 0.0882, with a repeatability of 0.1767 (Table 3); both fall into the ranges previously reported. Considerations to be taken into account when interpreting these varying estimates in the literature include impacts relating to the extent of variability (residual and genetic) in the populations sampled, the heritability calculation used, the trait definition, the prediction model used (animal/sire, linear/threshold), and study population used. Regardless of differences in approach, the results of this study corroborate previous reports, and the non-zero heritability estimate, alongside the evident genetic variation for TWIN in cattle populations signifies that inclusion of the trait in well-structured breeding strategies that will apply appropriate selection pressure will result in genetic progress toward lower risk of TWIN pregnancies in a given lactation.

There exists both qualitative and quantitative evidence of an association between twinning and other traits of (economic and wellness) importance in dairy production. Previous phenotypic associations reported implicate correlations between twinning and mortality, stillbirths and abortions, and reduced birth weights for calves [2,17,18,19]. For cows, higher incidences of periparturient diseases such as retained placenta, metritis, displaced abomasum, and ketosis, in addition to reduced productive lifespan for twin-bearing cows have been reported [2,20,21,22,23]. Additionally, associations have been reported between twinning and reproductive performance of cows and heifers alike, including increased days open, higher ovulation rates, increased services per conception, cystic ovarian disease and dystocia for cows, and freemartinism in most heifer calves twinned to bull calves [1,2,4,21,24,25].

In the present study, we reported genetic correlations between genomic predictions for TWIN and genomic predictions for an index and traits of importance for dairy wellness and profitability (Table 5 and Table 6). As expected, TWIN is positively correlated with other wellness traits pertaining to reproductive wellness: abortions (r = 0.25), retained placenta (r = 0.24) and metritis (r = 0.13), indicating that twinning is associated with other reproductive problems in dairy females. The highest correlation (r = 0.25) was observed with abortions, which was expected, as twin pregnancies often end in abortions. The correlations between TWIN and KETO and TWIN and DA are positive yet weak (0.05 and 0.02, respectively), suggesting that previous observations in the literature regarding such correlations between these health events could be influenced to a larger extent by environmental factors. Nevertheless, the non-zero correlation suggests there is a genetic association between these traits, albeit weak. As these traits are all expressed in STAs, positive correlations are interpreted as improvement in one will result in improvement of another. This indicates that applying selection pressure on these traits will result in genetic progress in the same direction for all (with the exception of LAME); in a similar vein, this also implies that cows that are more genetically predisposed to TWIN are more likely to experience abortion, retained placenta and metritis events. These correlations make sense physiologically, as the transition period for cattle is a stressful period, and is potentially exacerbated for twin-bearing cows. These correlations may also imply that these reproductive wellness traits are controlled by the overall reproductive system of the cow. The correlations of TWIN STAs with the CDCB trait PTAs are interpreted in the opposite manner—A positive correlation infers that improvement in a trait (g)PTA will result in a corresponding disimprovement in TWIN STA. All correlations with the traits in the US CDCB genetic evaluation were weak in nature. The strongest of these is the negative correlation between TWIN and PL (r = −0.055), which is not unexpected, given twinning has been previously associated with reduced productive lifespan in dairy cattle [23]. Thus, selecting for TWIN will result in indirect selection for a more favorable productive life. 

As with Ron et al. (1990) [35], the present study did not find an appreciable correlation between genomic predictions for TWIN and production traits (magnitudes ranging from 0.011 to 0.038 for milk, fat and protein; Table 6). Thus, selecting for reduced risk of TWIN will have little or negligible impact on making genetic progress for production traits. It is not unreasonable to surmise that previous reports implicating milk production and twinning being correlated could be underpinned by the many environmental and physiologic factors that influence twinning risk i.e., parity, season, ovulation rate, previous twin calving events, pharmaceutical use, hormonal fluctuations in high-feed intake, high-producing cows, plane of nutrition etc. [1,2,3,4,5,6,7,8,30]. In contrast to phenotypic associations in the literature, there is a weak to negligible correlation between genomic predictions for TWIN and cystic ovary, productive life, livability and the fertility traits (DPR, HCR and CCR) with magnitudes ranging from 0.011–0.031 (Table 6). The differences in magnitudes of correlations could also be due to differing models, populations sampled, data editing procedures and, simply, because the multifactorial nature of these quantitative traits results in environmental/physiological influences having a large impact on the expression of these traits. This offers the opportunity to use holistic and complementary management strategies (e.g., nutritional, pharmaceutical and breeding strategies) to reduce the relative risk in cattle experiencing these events. Furthermore, these negligible correlations showed that predictions for TWIN provides new information about an animal’s genetic potential for TWIN, further demonstrating the importance for its inclusion in a multitrait selection index that focuses on dairy wellness and profitability.

Most importantly, the correlation between TWIN and DWP$ was positive at 0.115, inferring selecting on DWP$ will result in genetic progress for TWIN, in parallel with all other trait categories in the index (cow and calf wellness, production, fertility, longevity, functional type, milk quality and calving). TWIN’s inclusion in the 2020 update of the DWP$ index was predicated on the fact that it was identified as a trait that impacts the lifetime profitability of a dairy female, and which occurs at an appreciable incidence in the industry. Subsequently, to quantify how selecting on DWP$ would alter genetic progress of its (aforementioned) underlying traits, the expected response to selection per standard deviation of genetic improvement of the index has been estimated previously (Zoetis data on file, Appendix A, Supplementary Materials S1). Inclusion of TWIN at a 1% weighting, coupled with a 1 standard deviation of genetic improvement on DWP$ index STA results in an expected response to selection of 0.81 STA (i.e., a lower relative risk of TWIN in a given lactation), alongside 218lbs of milk, 1.44 months of PL, 0.27% DPR, $9.47 Calving Ability, 0.80 STA for retained placenta and 0.55 STA for abortion. For further information on this response to selection, please refer to the Appendix A and the Supplementary Materials section.

### 4.2. (g)PTAs and (g)STAs of Twinning

To the best of our knowledge, this study is the first to report genomically enhanced PTAs ((g)PTAs) and STAs using a threshold animal model and ssGBLUP methodology for TWIN in Holstein dairy females. The means, standard deviations, and extreme values of gPTAs for TWIN are comparable to those previously obtained for other wellness traits in Holstein [61] and Jersey cows [64]. TWIN (g)PTAs reported in the present study range from −7.449–20.792%, with means ranging from −0.096–0.147% and SD ranging from 2.55–2.61%, depending on cohort evaluated (Table 4). As seen from Figure 1 (normal distribution curve), the numerical variation in (g)PTAs is considerable, and we have reported one of the widest ranges in (g)PTAs seen for TWIN. The most extreme positive (g)PTA value for twinning was obtained for a bull with 106 phenotyped daughters. Out of a total of 1,145,323 animals, 1,940 had a (g)PTA greater than 10. Generally, a broader range of (g)PTAs is preferable, because it enables better segregation of animals of different genetic merit for the trait. The average genomic reliability of about 40% for young genotyped animals without their own phenotypes or progeny is also comparable with genomic reliabilites for other similarly heritable traits. A total of 6 animals in the analysis had a reliability of zero, which may not be expected in genotyped animals. Zero or very low reliabilities are the artifact of the formula used for approximation of reliabilities, which relies on the diagonal values of the genomic relationship matrix (GRM). Animals that are not very well related to the majority of the population (e.g., crossbred animals), or animals genetically isolated from US Holstein genetics may have high values of the GRM diagonals, resulting in underestimation of reliabilities.

These present estimates are based on a large, longitudinal dataset which has undergone rigorous data editing procedures, and demonstrates wide-ranging variability in genomic merit within the study population. The inclusion of producer-recorded on-farm records shows that even with the potential intra-herd variation in phenotype recording (i.e., some herds potentially do not record, or record inconsistently given the obvious nature of the phenotype), the data editing and modeling approach employed yielded heritability and (g)PTA/STA estimates demonstrating exploitable variation, whilst reflecting actual on-farm situations. Moreover, the advantages of using an animal model lies in the fact that they are more suited to calculating variance components given that they account for all relationships amongst animals by taking in to account selection and assortative mating (i.e., accounts for both maternal and paternal influences) [73]. Furthermore, using cutting-edge ssGBLUP methodology for genomic evaluations removes risk of double counting and reduces pre-selection bias, resulting in improved accuracy of selection for low heritability traits and traits with incomplete information [61,74,75]. This prediction supports the much-needed shift in the industry from an historically reactive treatment to a proactive preventative to combat the rise in twinning incidences in dairy herds across the globe.

We converted the TWIN (g)PTAs to STAs for ease of interpretation and thus to facilitate ease of selection decisions. As McNeel et al. (2017) [54] outlined, a value of 100 represents the average expected TWIN risk, with the standard deviation being 5. STAs greater than 100 represent lower expected average disease risk relative to the base population in this study. Thus, higher STAs are more desirable, and selecting for a higher TWIN STA value will apply selection pressure for reduced genetic risk of TWIN. Genetic progress and thus adaptability in the long-term relies on ample exploitable genetic variation in the population, which has been demonstrated in the present study [76].

### 4.3. Demonstration of Evaluation Efficacy

In the current study, we hypothesized that cows with the highest genetic risk (lowest STAs) for TWIN would have a higher phenotypic incidence of TWIN than cows with the lowest genetic risk (highest STAs). As outlined by McNeel et al. (2017) [54], an accepted best practice for any genetic evaluation is to evaluate the association of its genetic predictions with the observed phenotypes of the evaluated animals in an external population. To our knowledge, this is the first time such a demonstration has been completed for genetic(omic) predictions for twin pregnancies. The results of the current study demonstrate the ability of the TWIN prediction to accurately predict TWIN incidence in a given lactation. Given the negligible incidence of twinning in the 1st lactation for all 5 herds (Table 7), the analysis was restricted to 2nd–4th lactation. This trend has been demonstrated and outlined previously [30]. 

**Figure 2:**
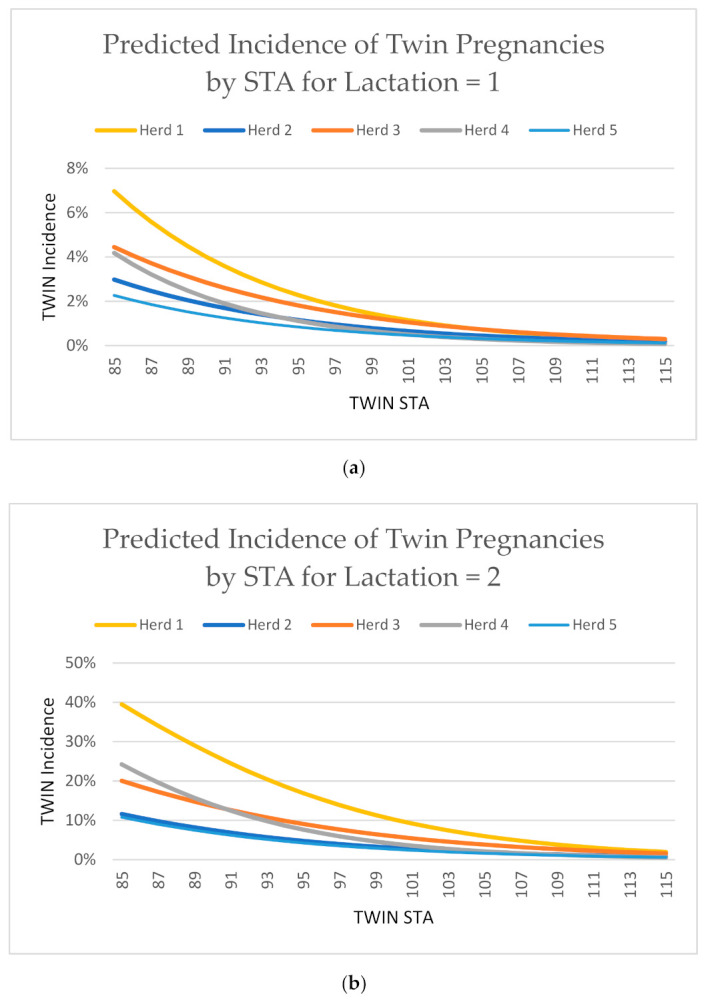

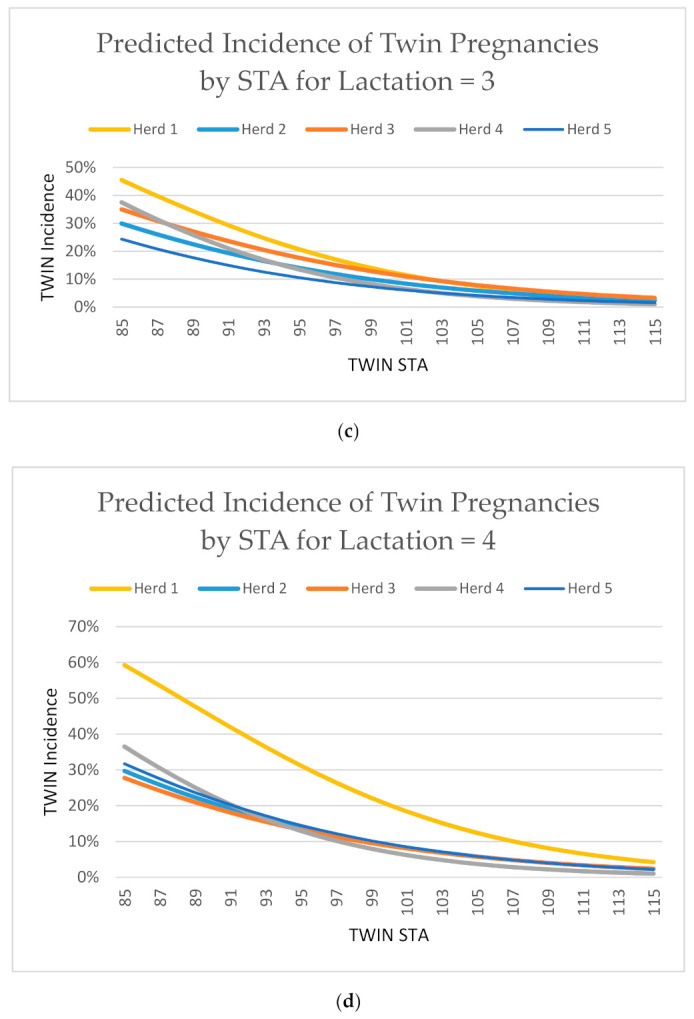
(**a**–**d**) illustrates the association between TWIN incidence and individual females ranked by TWIN STAs on a per lactation basis (1st–4th), for each herd. As expected, as the TWIN STA increases (i.e., the expected risk of a Holstein female of being pregnant with twins in a given lactation decreases), so too does the TWIN incidence, across all herds, all reproduction protocols, and across all lactations.

The association between individual TWIN STAs and twinning incidence illustrated in Figure 2 demonstrate that as expected, when STA values increase, twinning incidence decreases across all herds, all reproductive protocols, and all lactations. Unsurprisingly, the herd that used no synchronization protocol (herd 1) had the highest twinning incidence of all herds regardless of lactation, and those that used a synchronization protocol had a relatively lower incidence, which corroborates previous reports that Double Ovsynch protocols reduce twinning incidence on farm [30] and provides evidence that using genetics and management practices in tandem can help reduce on farm twinning incidences. Although some studies have previously reported seasonal effects on the incidence of twinning [2,3], inclusion of a season of conception effect in the model in the present study did not contribute to explaining variation in twinning incidences/events, given both TWIN STA and peak lactation output for the previous lactation were included in the model. This lack of association reported here mirrors results reported previously [1]. Peak lactation was significant for one of the five farms, which corroborates previous studies [5,77]. However, TWIN STA is the most consistently significant predictor of twinning incidence on farm, as demonstrated in the present study. 

This demonstration shows that producers can use the TWIN prediction to effectively reduce twinning incidence on-farm, with associated economic benefits (as reported in Table 8—up to a 78% reduction in frequency of twinning, with up to $15 in reduced cost losses per cow can be achieved). In future, when sufficient record numbers from herds representing a global cohort of females are accumulated, it could be beneficial to conduct another demonstration study on a superset of consolidated data with reproductive protocol included as a fixed effect in the model. In any case, as outlined by Van Vleck et al. (1991 (b)) [52], genetic evaluations are useful for early selection as producers to not need to have parturition records to make decisions based on these predictions. In the present study, we have corroborated this observation through the demonstration presented here. Thus, direct selection on TWIN can play an important role in reducing twinning incidences in Holstein populations, again, provided it is part of a holistic breeding and management strategy that selectively targets appropriate replacement heifers according to the producer’s breeding objective(s).

### 4.4. Inclusion in Index & Breeding Strategies to Complement Current Management Strategies

As outlined by Fessenden et al. (2020) [56], selection indices are a crucial component of many breeding programs, and are designed to facilitate selection for balanced genetic improvement across multiple traits of economic and production importance to producers [56,57]. Thus, inclusion of TWIN in a selection index is warranted, and in the present study TWIN was included in the DWP$ index. Defining practical ways to incorporate this information into a holistic breeding and management strategy will empower producers to extract the maximum value from the body of knowledge that exists today on twinning in dairy cattle. Any consideration for breeding for higher TWIN STAs (and thus lower risk of TWIN) should be undertaken within the framework of a multitrait breeding objective to account for relationships between traits and avoid unintended consequences [78]. Selection indices facilitate multitrait breeding objectives to be realised (once appropriate selection pressure is applied to the population) by providing a tool for producers to rank and select animals based on a single value that incorporates genetic merit information from many traits [55].

To our knowledge, this is the first time a prediction for TWIN has been included in a multitrait selection index for the dairy industry, which constitutes a novel step forward when it comes to harnessing genetic(omic) technologies in a practical way for producers to proactively reduce TWIN in Holstein populations. Previous genetic(omic) merit estimates generated for TWIN served other purposes such as (1) demonstrating variation in PTAs in cattle populations, (2) identifying QTLs associated with twinning rates and (3) inclusion as an input for genome-wide association studies [16,32,35,37,39,40]. The objective of this study was to include it in a selection index to empower producers in making sound breeding and management decisions to proactively reduce twinning risk.

This empirical theoretical and practical evidence demonstrating the exploitable genetic component to TWIN in Holsteins offers a solution for reducing TWIN in dairy cattle populations. One major advantage of this genetic component is that genetic progress made upon selection is cumulative, and can be permanent provided it is consistently selected for by producers. Environmental influences that can impact TWIN risks are numerous and have been outlined by Fricke (2001), Fricke (2015), López-Gatius et al. (2017) and Mur-Novales et al. (2018) [11,12,27,30]. Opinions differ on what is the optimal way to manage females carrying twin calves, with some advocating manual embryo reduction [27], and others suggesting selective culling and selective maintenance of pregnancy depending on the value of the cow and calf(ves) as the preferred alternative [11,12]. Another important strategy for producers to consider employing on-farm is hormonal manipulation of dairy females using a Double Ovsynch protocol before artificial insemination to reduce the likelihood of double ovulation [30]. The physiological basis for this strategy is that the protocol increases progesterone during follicular development which results in a reduced incidence of double ovulation and subsequent dizygotic twinning. Importantly, this strategy was the first non-genomic (i.e., environmental) management strategy proposed that proactively mitigates twinning risk in dairy females, and thus should be included in a holistic breeding and management approach to mitigate twinning risks on-farm.

All methods previously proposed have merit and their various combinations should be employed depending on the specific circumstances of each herd e.g., resource availability and allocation (e.g., for purchasing semen from high index and TWIN STA bulls, purchasing therapeutics for Double Ovsynch protocols etc.). An important consideration is that the positive effects of environmental management decisions to reduce twinning events are short-lived considering they impact the animal today and cannot be transmitted to the next generation, whereas the genetic(omic) merit of a cow is a function of all selection decisions that were made throughout her ancestral generations [78]. TWIN is a multifactorial trait, and should be treated as such when designing breeding and management strategies to reduce in on herd. Hence, a truly effective strategy to reduce TWIN in dairy populations depends on the complementary combination of both environmental and genetic management strategies to balance mitigation of TWIN risk in both a short-term and long-term fashion. Given previous limitations regarding lack of selection tools available to producers, the results described in the present study provide additional options for producers to best manage their females in terms of selecting females for reduced TWIN risk, and also informing management decisions for twin-bearing cows. Previously, Fricke (2015) [30] has proposed a two-pronged approach including (1) using a Double Ovsynch protocol before artificial insemination, and (2) selective reduction of unilateral twins/selective maintenance of cows with bilateral twin pregnancies. Lopez-Gatius (2020) [31] outlined using gonadotrophin-releasing hormone treatment for pregnancy maintenance and embryo reduction preferentially using PGF2 depending upon multiple factors such as genetic merit and stage of lactation. We propose a hybrid approach in which genetic merit information is utilized to differentially inform management decisions for cows, using a selection index value as the first consideration, followed by the TWIN trait merit, and subsequent management protocols. 

The first step of a genomically enhanced management strategy involves genomically testing all females in the herd, with the aim to select on indexes that include TWIN predictions (such as DWP$). Applying an appropriate level of selection pressure based on the index merit values (e.g., retain the top 80% of females every year, provided potential changes to the herd’s parity structure is economically viable) should result in genomic progress for TWIN along with production and wellness traits thus breeding a robust cow. Sire selection should consider the genomic merit for DWP$ and TWIN (high STAs) in order to minimize TWIN pregnancies. Coupled with Double Ovsynch protocols, this strategy should further reduce the incidence on TWIN on-farm. Optionally, the top 80% females retained annually on DWP$ could then be secondarily ranked on TWIN, and Double Ovsynch strategies employed for those with the lowest TWIN STAs, and/or those with the lowest STAs are only bred to bulls of a high TWIN STA merit (followed by sufficient early detection of TWIN via transrectal ultrasonography to inform future management decisions). In line with recommendations suggested by Lopez-Gatius (2020) [31], cows with a high index merit that are carrying twin calves may be candidates for pregnancy maintenance. However, given that previous twinning is a risk factor for future twinning events, such cows should ideally be bred to high index and TWIN merit bulls, after a DoubleOvsynch protocol implementation. In essence, a ‘coevolution’ of breeding and management strategies is required to establish a short-term and long-term sustainable solution to twinning in dairy herds. The TWIN and DWP$ predictions are a useful tool for dairy producers interested in using genetic(omic)ally enhanced strategies to improve their overall herd twin pregnancy incidence and profitability.

Our evaluation results are based on over one million producer-recorded twinning records, and over one million genotypes. Continuous data collection and inclusion of both new data from existing herds and historical data from new herds will increase the sampling depth in the Holstein population, simultaneously allowing for the variation in (g)PTA estimates observed to converge toward the true population variation, and reliability of records to increase given sufficient time. A concurrent and complementary approach when combining genetic(omic) tools and reproductive protocols ushers in the new era of precision management of dairy cattle, which should extract the maximum value of genetic(omic) predictions in relation to improving dairy wellness and herd sustainability.

## 5. Conclusions

This study showed that on-farm, producer-recorded twinning data can be successfully used in routine genetic and genomic evaluations for Holstein cattle. Moreover, the twinning predictions outputted by the evaluation have been demonstrated to effectively identify cows that are more genetically predisposed to experiencing twin pregnancies (regardless of reproduction protocol) when they enter the milking herd. Genetic correlations with Zoetis dairy reproduction wellness traits were low yet positive and indicate that they are controlled by the overall reproductive system of the cow. Successful inclusion of this trait in to a multitrait selection index whose aim is to improve the overall herd wellness and profitability offers a new selection tool for dairy reproduction and wellness improvement which was not available to producers until now. Using such a tool to drive on-farm genetic selection for improved reproduction and wellness traits will confer cumulative improvement of a herd’s reproductive and health status. In the shorter-term temporary relief offered by other management interventions (therapeutic or otherwise) currently available today can be used in tandem. Moreover, concurrent management interventions can complement genetic selection strategies to further reduce the risk/incidence of twinning in the herd concomitantly. The inclusion of novel reproduction traits such as twinning in a selection index offers producers a more comprehensive tool for selecting potentially more robust, sustainable and profitable animals.

## Figures and Tables

**Table 1:** Standardization and collapsing of on-farm terminology used to record TWIN events analyzed in this study. All such combinations were collapsed into TWIN (1). The absence of these combinations resulted in the cow being classified as having a standard, singleton pregnancy (0).

Event	Remark	Standardized Event
ABORT	TWIN, TW M, TW F, TW=, TWF, TWDOA, TWM, BULHFRTW, TW2BULL, TWNB, TWNH, BULLSTW, TWNHFRS, TWBULL, TW (and remark is only two letters long)	TWIN
RP		TWIN
MISC		TWIN
DRYOFF		TWIN
ILL		TWIN
OK		TWIN
REMARK		TWIN
SOLD		TWIN
FRESH		TWIN

ABORT = abortion; RP = retained placenta; MISC = miscellaneous; DRYOFF = female was dried off; ILL = female’s health status was recorded as ill; OK = health status recorded as normal; REMARK = item describing the event further (e.g. treatment given, location of treatment, cause of event etc.); SOLD = female was sold; FRESH = female calved.

**Table 2:** Characteristics of the data in the genomic evaluation for twinning in Holsteins (August 2020).

Item	Count
Pedigree records total	3,687,609
Phenotypic records total	3,528,053
Animals with phenotypes	1,800,296
Animals with genotypes	1,145,323
Animals with genotypes and phenotypes	78,509
Incidence of twinning	3.25%

**Table 3:** Estimated variance components for twinning in Holsteins.

Trait	σ^2^_g_	σ^2^_pe_	σ^2^_hys_	σ^2^_e_	h^2^	r^2^
TWIN	0.1315	0.1318	0.2272	1.0	0.0882	0.1767

σ^2^_g_ = additive genetic variance; σ^2^_pe_ = permanent environmental variance; σ^2^_hys_ = HYS variance; σ^2^_e_ = residual variance; h^2^ = heritability; r^2^ = repeatability_._

**Table 4:** Summary statistics for (g)PTAs, STAs, and reliabilities for TWIN predictions as outputted by the genetic evaluation for (1) all Holsteins with genotypes, phenotypes and/or progeny and (2) Holsteins with genotypes only in the evaluation.

All Animals with Genotypes					
Variables *	*n*	Mean	Std Dev	Minimum	Maximum
TWIN PTA	1,145,323	−0.096	2.606	−7.449	20.792
TWIN STA	1,145,323	100.29	4.636	63	113
TWIN REL	1,145,323	42.02	6.144	0	99.5
Animals with genotypes (no phenotypes, no progeny)					
TWIN PTA	887,068	−0.147	2.550	−7.449	19.703
TWIN STA	887,068	100.38	4.537	65	113
TWIN REL	887,068	40.77	5.099	0	61.140

* TWIN PTA = (g)PTAs for twinning; TWIN STA = STAs for twinning; TWIN REL = reliabilities of (g)PTAs for twinning.

**Table 5:** Product-moment (Pearson) correlations between TWIN and Zoetis’ Holstein DWP$ index and wellness traits (*n* = 1,145,323).

			Zoetis Wellness Traits *											
Trait	DWP$	DIAR	CALF_RESP	DEAD	RETP	METR	MAST	LAME	KETO	DA	MFV	ABRT	RESP	CYST
TWIN	0.115	0.014	0.021	0.065	0.239	0.123	0.025	−0.025	0.045	0.024	0.029	0.252	0.013	0.056

* DIAR = calf scours; CALF_RESP = calf respiratory disease; DEAD = calf livability; RETP = retained placenta; METR = metritis; MAST = mastitis; LAME = lameness; KETO = ketosis; DA = displaced abomasum; MFV = milk fever; ABRT = abortion; RESP = cow respiratory disease; CYST = cystic ovaries. All correlations are based on Zoetis DWP$ and wellness traits being expressed in STAs (i.e., the directionality of the correlations are the same).

**Table 6:** Product-moment (Pearson) correlations between TWIN and CDCB traits (*n* = 1,076,031).

	CDCB Traits **								
Trait	Milk	Fat	Prot	PL	LIV	SCS	DPR	HCR	CCR
TWIN	0.011	−0.038	0.025	−0.055	−0.029	0.029	0.014	−0.031	−0.011

** Milk = milk yield; Fat = fat yield; Prot = protein yield; PL = productive life; LIV = cow livability; SCS = somatic cell score; DPR = daughter pregnancy rate; HCR = heifer conception rate; CCR = cow conception rate. All correlations are based on the CDCB traits being expressed in PTAs, and Zoetis TWIN being expressed as an STA (i.e., the directionality of the correlation is reversed).

**Table 7:** Overview of the 5 herds used in the demonstration cohort including reproductive protocol used, region of the US the farm is located, total number of females, total number of calving records, average TWIN STA, minimum TWIN STA, maximum TWIN STA, 1st lactation incidence of twinning and 3^rd^ lactation twinning incidence.

Herd	Protocol	Region	No. Females	No. Records	Average TWIN STA	Minimum TWIN STA	Maximum TWIN STA	1st Lactation TWIN Incidence (%)	3rd Lactation TWIN Incidence (%)
1	No synchronization	West	477	1483	100	88	108	1.48	14.00
2	Presynch/ovsynch	Mid-West	1105	3050	101	77	108	0.74	9.09
3	Presynch/ovsynch	Mid-West	2157	5434	100	86	110	1.25	12.93
4	Double Ovsynch	Mid-West	3200	8721	100	79	110	0.64	7.79
5	Double Ovsynch	North West	1280	3592	100	79	115	0.56	6.95

**Table 8:** Least squares means for TWIN STA genetic groups (*n* = 3), TWIN incidence (marginal means), SEM of the genetic groups when animals are ranked by TWIN STA within herd, *p*-value, and estimated TWIN cost per case (i.e., cow) for each herd.

Herd	STA Genetic Group	TWIN Incidence	SEM	*p*-Value	TWIN Cost Per Case ($)
1	Bottom 33%	0.19	0.027	<0.0001	31
	34–66%	0.14	0.024		23
	Top 33%	0.10	0.022		16
2	Bottom 33%	0.09	0.013	<0.0001	14
	34–66%	0.06	0.010		10
	Top 33%	0.04	0.009		6
3	Bottom 33%	0.12	0.011	<0.0001	19
	34–66%	0.09	0.009		14
	Top 33%	0.06	0.008		10
4	Bottom 33%	0.09	0.008	<0.0001	14
	34–66%	0.07	0.006		11
	Top 33%	0.03	0.004		5
5	Bottom 33%	0.09	0.012	<0.0001	14
	34–66%	0.06	0.008		10
	Top 33%	0.02	0.006		3

**Table 9:** Type III Tests of Fixed Effects in the model used for the tertiary analysis on 3rd lactation cows (regressing individual animal TWIN STA on twinning incidence), degrees of freedom, F-value and associated *p*-value. Fixed effects of TWIN STA, season of conception and peak lactation output from the previous lactation are reported.

Herd	Fixed Effect	df	F-Value	*p*-Value
1	TWIN STA 2013	1	7.68	0.0060
	Conception Season	3	0.18	0.9129
	Peak Lactation	1	0.02	0.8849
2	TWIN STA 2013	1	2.50	0.1147
	Conception Season	3	0.54	0.6563
	Peak Lactation	1	2.75	0.0981
3	TWIN STA 2013	1	7.64	0.0058
	Conception Season	3	0.77	0.5105
	Peak Lactation	1	3.87	0.0495
4	TWIN STA 2013	1	8.66	0.0033
	Conception Season	3	1.29	0.2779
	Peak Lactation	1	0.46	0.4981
5	TWIN STA 2013	1	4.73	0.0300
	Conception Season	3	0.58	0.6295
	Peak Lactation	1	3.42	0.0650

## Data Availability

Data supporting reported results can be found in the Supplementary Materials and of this manuscript.

## Supplementary Materials

The following are available online at https://www.mdpi.com/2076-2615/11/3/843/s1, Material S1: Building a Healthier Herd with CLARIFIDE^®^ PLUS, Technical Bulletin CLR-00428.

## Appendix A

**Table A1:** Expected response to selection expressed in units of the underlying trait when average DWP$ 2020 is increased by 1SD. Adapted from Zoetis data on file, Technical Bulletin CLR-00428, Table 2.

Trait	Expected Response to Selection
Fat (lbs)	15
Protein (lbs)	10
Milk (lbs)	218
Productive Life (mo.)	1.44
Cow Livability (%)	0.90
Somatic Cell Score (log)	−0.05
Body Size Composite (pts)	−0.22
Udder Composite (pts)	0.21
Feet & Leg Composite (pts)	0.10
Daughter Pregnancy Rate (%)	0.27
Heifer Conception Rate (%)	0.32
Cow Conception Rate (%)	0.52
Calving Ability ($)	9.47
Zoetis Mastitis (STA)	2.44
Zoetis Metritis (STA)	1.98
Zoetis Retained Placenta (STA)	0.80
Zoetis Displaced Abomasum (STA)	1.14
Zoetis Ketosis (STA)	2.04
Zoetis Lameness (STA)	1.22
Zoetis Calf Respiratory (STA)	1.16
Zoetis Calf Scours (STA)	1.16
Zoetis Calf Livability (STA)	1.46
Zoetis Cow Respiratory (STA)	1.35
Zoetis Cystic Ovary (STA)	0.26
Zoetis Twinning (STA)	0.81
Zoetis Cow Abortion (STA)	0.55
