# Supplementary material for: Genomic Prediction for Twin Pregnancies
Source: Animals (Basel). 2021 Mar 16;11(3):843. doi: 10.3390/ani11030843 (PMC8002547; doi:10.3390/ani11030843)
Supplement: Supplementary file 1 [file animals-11-00843-s001.pdf]

TECHNICAL BULLETIN

Building a Healthier Herd with CLARIFIDE® PLUS

Dairy producers can use CLARIFIDE Plus to select animals based on traits that affect health, performance and lifetime profit of cows and calves with a goal of a healthier, more productive herd.

Zoetis
10 Sylvan Way
Parsippany, NJ 07054

KEY POINTS

- Zoetis Genetics is updating Dairy Wellness Profit Index® (DWP$®) for Holstein in April 2020. Additional traits that impact lifetime profitability are now available, and the economic values of current traits within Dairy Wellness Profit Index have been updated to be representative of current and future prices.
- DWP$ 2020 has a standard deviation of 254DWP$, while DWP$ 2018 had a standard deviation of 242DWP$. By including more traits affecting profitability, DWP$ 2020 describes more genetic variation in profit than DWP$ 2018.
- The 2020 formulation of DWP$ applies increased genetic selection against abortion, twinning, cystic ovaries, cow respiratory disease, and cow size compared to the 2018 version of DWP$.
- The updates and incorporation of new fertility and wellness traits to DWP$ will increase DWP$'s ability to predict potential lifetime profitability.
- Comparing the predictive capacity of DWP$ 2020 against DWP$ 2018, the best 25% of cows was associated with 1142 more pounds of lifetime ECM and 141 USD in lifetime Income over Feed Costs per cow compared to the worst 25% of cows.[12] These results indicate that genomically enhanced DWP$ predictions for young calves can be used to effectively predict potential lifetime profitability.

Introduction

Selection indexes are a critical component of many breeding programs and provide a way to combine information about many traits into a single number that producers can use to rank animals and inform breeding decisions.[1] The purpose of a selection index is to predict an animal's genetic potential for total economic merit.[2] Historically, selection indexes throughout the world have focused on improving production traits.[3] However, this narrow selection goal contributed to a decrease in health and fertility.[4] Improvement of phenotype recording and development

of new trait evaluations led to fertility,[5] longevity,[6] milk quality,[7] and health traits[8] becoming available to breeders. Over the last 25 years, incorporation of these non-production traits in selection indexes has grown[9] as breeders strive to account for both incomes and expenses of an animal.

Selection indexes such as Dairy Wellness Profit Index (DWP$), Wellness Trait Index® (WT$®), and Calf Wellness Index™ (CW$™) are key components of the CLARIFIDE Plus genomic test offering. Dairy producers may seek to improve their herds by genomic testing heifers and ranking them based upon DWP$ to inform culling and breeding decisions. The dairy industry benefits from genomic technology as it results in faster genetic progress.[10, 11] As the rate of genetic progress is increased, it becomes more important that DWP$ is correctly formulated and includes key economically important traits for the goal of lifetime profit. Dairy producers are leveraging genomic technology to increase selection intensity on their females through culling decisions very early in life and advanced reproductive technologies. With this combination of technologies and strategies, the costs for having a non-optimal selection index could be substantial.

As new traits become available, their influence on lifetime profitability is assessed and added to DWP$ when appropriate. Therefore, Zoetis updated DWP$ in April 2020 to incorporate new traits and update the economic values of traits previously in DWP$.

Dairy Wellness Profit Index Updates

Dairy Wellness Profit Index is a multi-trait selection index that includes cow and calf wellness, production, fertility, functional type, longevity, livability, calving ability, and milk quality traits plus polled test results. Developed in 2016 by Zoetis Genetics and updated in 2018, DWP$ estimates the potential lifetime profit an animal will contribute to the dairy operation. DWP$ is expressed in a dollar value with higher positive numbers indicating the animal has the genetic potential to generate and transmit more profit over her lifetime.

In the Dairy Wellness Profit Index 2020 update, numerous changes are being implemented:

- New traits that impact lifetime profitability including cow abortion, twinning, cow respiratory disease, and cystic ovaries and are included in the 2020 formulation of DWP$.
- As additional traits that impact the cow's ability to stay in the herd have become available, these traits are directly accounting for the impact of fertility and disease upon longevity. This is resulting in the economic value of productive life decreasing.
- Feet & Leg Composite and Udder Composite, both indirect predictors of Productive Life that are measured in first lactation, have been removed from DWP$ 2020. The lifetime validation study demonstrated that Feet & Leg Composite and Udder Composite did not improve the index; this may be due to both the continued genetic progress for confirmation and the inclusion of the Zoetis wellness traits.
- The economic values of fat and protein have increased. This was done to ensure that the economic values used to develop DWP$ are representative of current and future prices dairy farmers may experience.
- The negative economic value placed upon body weight composite has changed to better represent the feed cost of larger dairy cows.

As a result, the 2020 formulation of DWP$ describes more genetic variation compared to the 2018 version as documented by the large standard deviation (254 vs 242DWP). By including more traits affecting profitability, DWP$ 2020 describes more genetic variation in profit than DWP$ 2018.

To further understand the impact of these updates to DWP$, a side by side comparison of the emphasis placed upon each trait in DWP$ 2020 and DWP$ 2018 is available in Table 1.

The following charts show the amount of emphasis each general category included in DWP$ 2020 and DWP$ 2018.

To assess how the use of DWP$ 2020 would alter genetic progress of underlying traits when compared to DWP$ 2018, the expected response to selection per standard deviation of genetic improvement of the index was estimated. In examining the response of selection between DWP$ 2020 and DWP$ 2018, use of DWP$ 2020 will result in genetic improvement in abortion, twinning, cystic ovary, and cow respiratory. DWP$ 2020 has stronger selection against body size composite resulting in selection for a more moderate sized cow. DWP$ 2020 will maintain a similar selection response for the rest of the traits in DWP$ (Table 2).

Association Between DWP$® Predictions and Lifetime Performance

As some of the first heifers tested by Zoetis are now finishing their careers, we are now able to examine how well DWP$ predicted lifetime profit. In order to determine if DWP$ 2020 improves the capability to predict lifetime profitability better than DWP$ 2018 a side by side comparison was conducted.[13]

Five large herds (n=2,175 enrolled cows) in the United States were chosen for this study because they had: (1) genomic predictions from females born in 2011, (2) recorded production, reproduction and health events to accurately estimate profit per cow (3) at least 200 animals born in 2011.

Dairy Wellness Profit (DWP$) predictions from 2012 were used to rank the 2,175 animals within herd and assign cows to percentile-based DWP$ groups (genetic groups: Worst 25%, 26–50%, 51–75%, and Best 25%).

Table 1 – Defines the relative values (%) for underlying traits for Dairy Wellness Profit 2020 and Dairy Wellness Profit 2018.

Trait	DWP$ 2020*	DWP$ 2018*
Fat	18	16
Protein	16	14
Milk	1	2
Productive Life	6	9
Cow Livability	3	4
Somatic Cell Score	-2	-3
Body Size Composite	-10	-3
Udder Composite	0	5
Feet & Legs Composite	0	2
Daughter Pregnancy Rate	3	4
Calving Ability	2	3
Heifer Conception Rate	1	1
Cow Conception Rate	1	1
Zoetis Mastitis	11	12
Zoetis Metritis	3	4
Zoetis Retained Placenta	1	1
Zoetis Displaced Abomasum	1	2
Zoetis Ketosis	<1	<1
Zoetis Lameness	5	6
Zoetis Calf Respiratory	2	2
Zoetis Calf Scours	2	2
Zoetis Calf Livability	2	4
Zoetis Cow Respiratory	1	0
Zoetis Cystic Ovary	1	0
Zoetis Cow Abortion	5	0
Zoetis Twinning	1	0

*Sum of absolute values equals 100%.

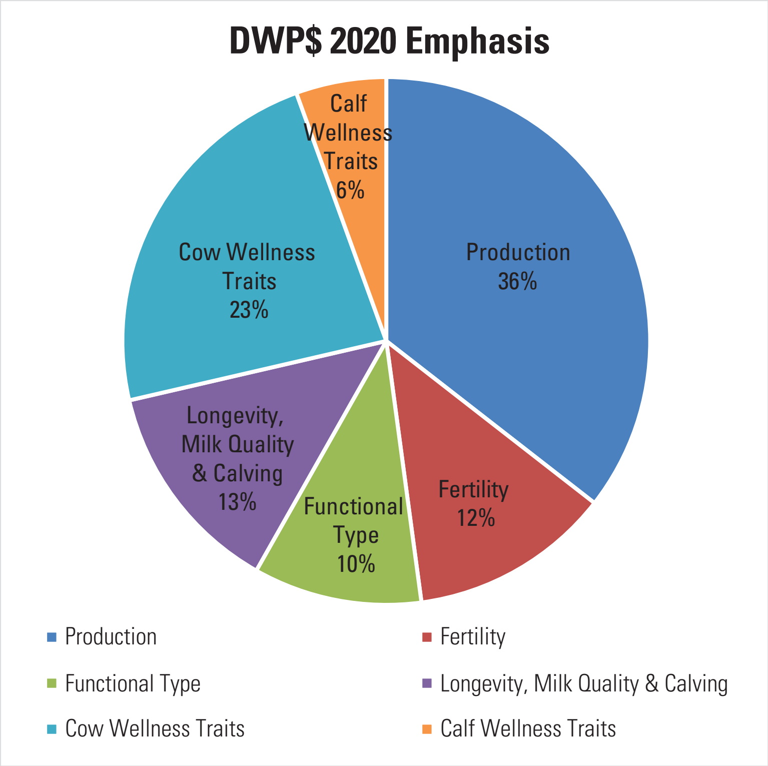

Chart 1 – DWP$ 2020 Emphasis (%).

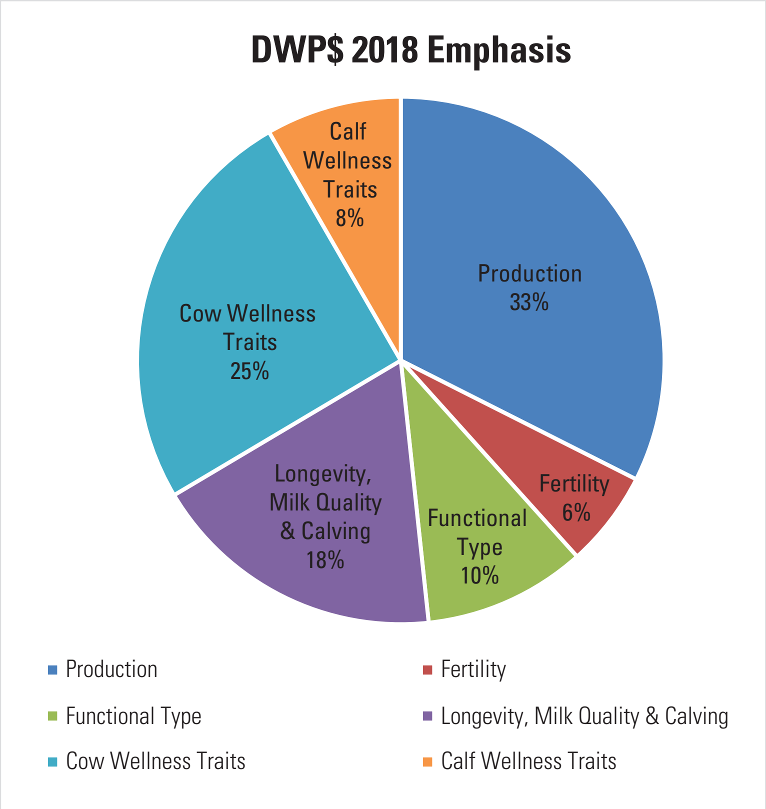

Chart 2 – DWP$ 2018 Emphasis (%).

Herd records were used to calculate lifetime energy corrected milk (ECM) and income over feed cost based on the actual performance from first freshening through when they left the herd; for cows that were still in the herd, current totals were used.

Table 3 shows that when ranked by DWP$ 2020, the best 25% of females produced 21,460 pounds more lifetime ECM per cow than the worst 25%. This additional lifetime ECM represents $1,428 additional Income Over Feed Cost (IOFC) per cow in the best 25% of females than the worst 25%. Table 4 shows that when ranked by DWP$ 2018, the best 25% of females produced 20,018 pounds more lifetime ECM per cow than the worst 25%. This additional lifetime ECM represents $1,287 additional Income Over Feed Cost (IOFC) per cow in the best 25% of females than the worst 25%. The larger difference in lifetime ECM and lifetime IOFC between the best and worst DWP$ groups when ranked by DWP$ 2020 indicates that DWP$ 2020 has improved the ability to predict potential lifetime profitability when compared with DWP$ 2018. The updates and the incorporation of the new fertility and wellness traits to DWP$ 2020 are increasing DWP$'s ability to predict potential lifetime profitability.

Use of DWP$ under real-world conditions confirms the value of DWP$ in helping to coordinate selection toward greater profitability. The 2020 update to DWP$ can assist dairy producers in their goal to improve overall lifetime profitability of their dairy herd.[12]

Table 2 – Expected response to selection expressed in units of the underlying trait when average DWP$ 2020 and DWP$ 2018 are increased by one standard deviation.

Trait	DWP$ 2020	DWP$ 2018
Fat (lbs)	15	15
Protein (lbs)	10	10
Milk (lbs)	218	224
Productive Life (mo.)	1.44	1.49
Cow Livability (%)	0.90	0.86
Somatic Cell Score (log)	-0.05	-0.06
Body Size Composite (pts)	-0.22	-0.12
Udder Composite (pts)	0.21	0.32
Feet & Leg Composite (pts)	0.10	0.17
Daughter Pregnancy Rate (%)	0.27	0.26
Heifer Conception Rate (%)	0.32	0.32
Cow Conception Rate (%)	0.52	0.50
Calving Ability ($)	9.47	10.17
Zoetis Mastitis (STA)	2.44	2.64
Zoetis Metritis (STA)	1.98	1.86
Zoetis Retained Placenta (STA)	0.80	0.60
Zoetis Displaced Abomasum (STA)	1.14	1.08
Zoetis Ketosis (STA)	2.04	2.10
Zoetis Lameness (STA)	1.22	1.17
Zoetis Calf Respiratory (STA)	1.16	1.28
Zoetis Calf Scours (STA)	1.16	1.31
Zoetis Calf Livability (STA)	1.46	1.47
Zoetis Cow Respiratory (STA)*	1.35	1.22
Zoetis Cystic Ovary (STA)*	0.26	0.03
Zoetis Twinning (STA)*	0.81	0.52
Zoetis Cow Abortion (STA)*	0.55	-0.05

*These traits were not included in DWP$ 2018. The genetic progress is due to the relationship between these traits and traits directly selected for in DWP$ 2018.

Table 3 – Association between lifetime performance and DWP$ 2020 genomic ranking.

CLARIFIDE Plus DWP$ Ranking	DWP$ GPTA Value ($)	Lifetime ECM (lbs.)	Lifetime IOFC per cow ($)	Lifetime Days in Milk (days)
76-100% (Best)	453	75,558[a]	4,813[a]	885[a]
51-75%	282	69,155[b]	4,344[b]	833[b]
25-50%	165	63,879[c]	4,033[c]	780[b]
0-25% (Worst)	-19	54,098[d]	3,385[d]	680[c]
Difference between Best & Worst	472	21,460	1,428	205

[a-d] Least Squares Means within column and DWP$ ranking with different superscripts diff ($P < 0.05$).

Table 4 – Association between lifetime performance and DWP$ 2018 genomic ranking.

CLARIFIDE Plus DWP$ Ranking	DWP$ GPTA Value ($)	Lifetime ECM (lbs.)	Lifetime IOFC per cow ($)	Lifetime Days in Milk (days)
76-100% (Best)	430	74,753[a]	4,733[a]	885[a]
51-75%	282	68,621[b]	4,318[b]	826[b]
25-50%	174	64,518[b]	4,074[b]	784[b]
0-25% (Worst)	3	54,735[c]	3,446[c]	683[c]
Difference between Best & Worst	427	20,018	1,287	202

[a-c] Least Squares Means within column and DWP$ ranking with different superscripts diff ($P < 0.05$).

Wellness Trait Index Update

To support selection for reduced risk of disease in dairy females, Zoetis updated the Wellness Trait Index® (WT$®). The 2020 update to WT$ is the inclusion of cow respiratory disease while continuing to include mastitis, lameness, metritis, retained placenta, displaced abomasum, and ketosis plus polled test results. The WT$ index directly estimates potential profit contribution of the wellness traits for an individual animal. By including more wellness traits that affect profitability, WT$ 2020 describes more genetic variation in profit with a standard deviation of 108WT$.

Summary

The 2020 updates to Dairy Wellness Profit and the Wellness Trait index provide an opportunity for dairy producers to continue to select for overall herd profitability. The side by side comparison of DWP$ 2020 and DWP$ 2018 demonstrate the ability of DWP$ predictions to predict lifetime profit of Holstein animals. These results indicate that DWP$ 2020 predictions for young calves can be used to predict potential lifetime profitability. Dairy Wellness Profit predictions are a useful tool for dairy producers interested in using genetics as a method to improve their overall herd profitability. Incorporating DWP$ 2020 into breeding and culling decisions will help dairy producers create future generations of animals that have the capability for higher lifetime profit when combined with best management practices.

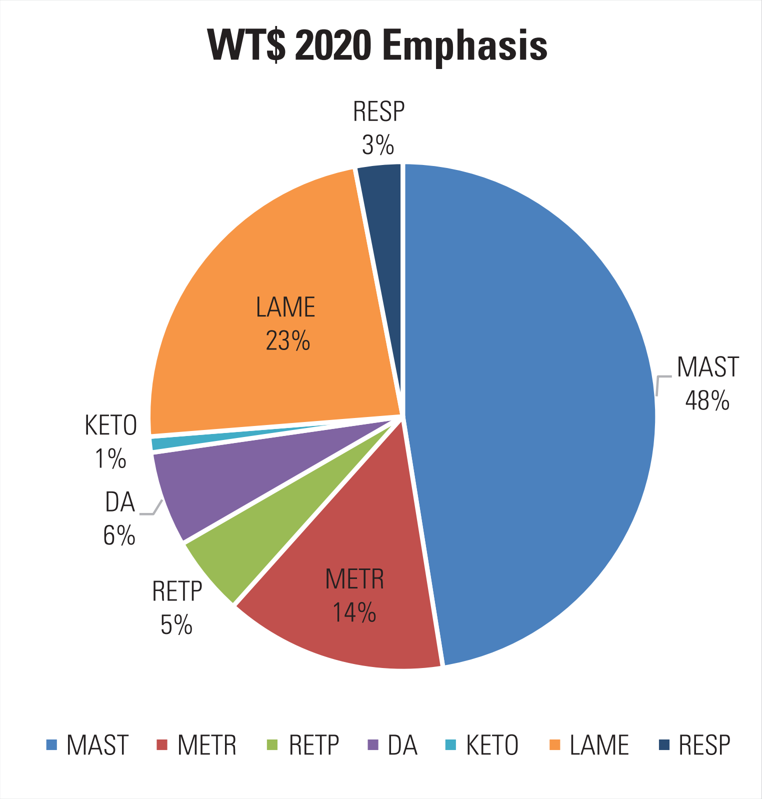

Chart 3 – WT$ 2020 Emphasis (%).

References

1. Cole, J. and P. VanRaden. *Possibilities in an age of genomics: The future of selection indices 1*. Journal of Dairy Science, 2018: p. 1-16.
2. Shook, G. *Major advances in determining appropriate selection goals*. Journal of Dairy Science, 2006. 89(4): p. 1349-1361.
3. Byrne, T., et al. *New breeding objectives and selection indices for the Australian dairy industry*. Journal of Dairy Science, 2016. 99(10): p. 8146-8167.
4. VanRaden, P. *Invited review: Selection on net merit to improve lifetime profit*. Journal of Dairy Science, 2004. 87(10): p. 3125-3131.
5. VanRaden, P., et al. *Development of a national genetic evaluation for cow fertility*. Journal of dairy science, 2004. 87(7): p. 2285-2292.
6. VanRaden, P. and G. Wiggans. *Productive life evaluations: Calculation, accuracy, and economic value*. Journal of dairy science, 1995. 78(3): p. 631-638.
7. Schutz, M.M. *Genetic evaluation of somatic cell scores for United States dairy cattle*. Journal of Dairy Science, 1994. 77(7): p. 2113-2129.
8. Vukasinovic, N., et al. *Development of genetic and genomic evaluation for wellness traits in US Holstein cows*. Journal of dairy science, 2017. 100(1): p. 428-438.
9. Miglior, F., B. Muir, and B. Van Doormaal. *Selection indices in Holstein cattle of various countries*. Journal of dairy science, 2005. 88(3): p. 1255-1263.
10. García-Ruiz, A., et al. *Changes in genetic selection differentials and generation intervals in US Holstein dairy cattle as a result of genomic selection*. Proceedings of the National Academy of Sciences, 2016. 113(28): p. E3995-E4004.
11. Hill, W.G. *Is continued genetic improvement of livestock sustainable?* Genetics, 2016. 202(3): p. 877-881.
12. Di Croce, F.D., et al. *Associations Between Dairy Wellness Profit Index®(DWP®) Predictions and Lifetime Performance*, 2019.
13. Data on File, Zoetis, July 2019.

All trademarks are the property of Zoetis Services LLC or a related company or a licensor unless otherwise noted.
© 2020 Zoetis Services LLC. All rights reserved. CLR-00428
